# Cardiovascular Physiology During Mechanical Circulatory Support: Implications for Management and Monitoring

**DOI:** 10.3390/jcm14196935

**Published:** 2025-09-30

**Authors:** Ettore Crimi, Karuna Rajkumar, Scott Coleman, Rohesh Fernando, Bryan Marchant, Chandrika Garner, John Gaillard, Megan H. Hicks, Ryan C. Maves, Ashish K. Khanna

**Affiliations:** 1 Department of Anesthesiology, School of Medicine, Wake Forest University, Winston-Salem, NC 27157, USA; karuna.putturrajkumar@advocatehealth.org (K.R.); scott.coleman@advocatehealth.org (S.C.); rohesh.fernando@advocatehealth.org (R.F.); bryan.marchant@advocateealth.org (B.M.); chandrika.garner@advocatehealth.org (C.G.); john.gaillard@advocatehealth.org (J.G.); megan.hicks@advocatehealth.org (M.H.H.); ryan.maves@advocatehealth.org (R.C.M.); ashish.khanna@advocatehealth.org (A.K.K.); 2 Department of Emergency Medicine, School of Medicine, Wake Forest University, Winston-Salem, NC 27157, USA; 3 Department of Internal Medicine, Section of Infectious Diseases, School of Medicine, Wake Forest University, Winston-Salem, NC 27157, USA; 4 Center for Bioethics, Health, and Society, Wake Forest University, Winston-Salem, NC 27109, USA; 5 Outcomes Research Consortium, Houston, TX 77030, USA

**Keywords:** mechanical circulatory support, cardiogenic shock, heart failure, hemodynamic monitoring, artificial intelligence

## Abstract

**Background/Objectives:** Mechanical circulatory support (MCS) is increasingly utilized for the management of acute decompensated heart failure (HF) and cardiogenic shock (CS). The primary goals of MCS are to restore systemic perfusion, reduce cardiac workload, and support end-organ function. A thorough understanding of cardiovascular physiology in patients supported by MCS is essential for clinical decision-making. This review summarizes current evidence on the physiological effects of various MCS devices, key monitoring techniques, patient management, and explores the emerging role of artificial intelligence (AI) in this field. **Main Text:** Short-term MCS devices include intra-aortic balloon pumps (IABP), percutaneous left-sided devices such as Impella (Abiomed, Danvers, MA, USA) and TandemHeart (LivaNova, London, UK), percutaneous right-sided support devices like Protek Duo (LivaNova, London, UK) and Impella RP Flex (Abiomed, Danvers, MA, USA), and veno-arterial extracorporeal membrane oxygenation (VA-ECMO). Long-term support is mainly provided by left ventricular assist devices (LVADs), including the HeartMate 3 (Abbott Laboratories, Chicago, IL, USA). Optimal MCS application requires an understanding of device-specific cardiovascular interactions and expertise in appropriate monitoring tools to assess device performance and patient response. The choice of device, timing of initiation, and patient selection must be individualized, with careful consideration of ethical implications. The integration of AI offers significant potential to advance clinical care by improving complication prediction, enabling real-time optimization of device settings, and refining patient selection criteria. **Conclusions:** MCS is a rapidly evolving field that requires a comprehensive understanding of cardiovascular interactions, careful selection of monitoring strategies, and individualized clinical management. Future research should address current device limitations, clarify device-specific clinical applications, and assess the validity of AI-driven technologies.

## 1. Introduction

Mechanical circulatory support (MCS) is increasingly used in the multidisciplinary management of decompensated heart failure (HF) and cardiogenic shock (CS). These complex clinical syndromes are characterized by impaired end-organ perfusion and high mortality rates ranging from 30% to 50% [1,2,3,4]. The primary goals of MCS are to restore systemic perfusion, decrease cardiac workload and myocardial oxygen demand, support end-organ function, and minimize prolonged high-dose inotropic support, which may exacerbate organ dysfunction [5]. Applications of MCS devices span from acute short-term stabilization to longer-term support aimed at bridging patients to myocardial recovery, transplantation, or destination therapy. Timely and individualized implementation of MCS strategies is critical to prevent progression of end-organ dysfunction and improve clinical outcomes [6,7,8,9]. A thorough understanding of cardiovascular physiology associated with MCS devices is essential for optimizing clinical management, patient selection, and monitoring strategies. This review describes the distinct physiological effects of each MCS device, highlights key invasive and non-invasive monitoring techniques, examines clinical management and ethical considerations, and explores the role of artificial intelligence in optimizing MCS strategies.

## 2. Cardiovascular Physiology in the Setting of MCS

### 2.1. Definitions of Heart Failure and Cardiogenic Shock

HF is a clinical syndrome resulting from structural and/or functional cardiac abnormalities that impair ventricular filling or ejection. HF is commonly categorized as HF with reduced ejection fraction (HFrEF), defined as left ventricular ejection fraction (LVEF) ≤40%; HF with mildly reduced ejection fraction (HFmrEF), defined as LVEF 41–49% and sharing characteristics of both reduced and preserved phenotypes; and HF with preserved ejection fraction (HFpEF), defined as LVEF ≥50% with objective evidence of increased left ventricular filling pressures such as elevated natriuretic peptides or abnormal hemodynamic indices [1]. The ACC/AHA stages of HF outline disease progression. Stage A identifies patients at risk for HF but without symptoms or structural abnormalities. Stage B (Pre-HF) refers to those with asymptomatic structural changes, abnormal biomarkers, or elevated filling pressures. Stage C is defined by structural heart disease with current or prior symptoms of HF. Stage D (advanced or end-stage HF) includes severe, refractory symptoms that interfere with daily life, recurrent hospitalizations, and poor prognosis despite optimal medical and device-based therapy [1].

End-stage HF (Stage D) represents advanced heart failure that is refractory to standard, guideline-directed medical and device-based therapies [10]. Patients in this category frequently require advanced interventions such as durable mechanical circulatory support (MCS) or cardiac transplantation. To further stratify advanced HF, the INTERMACS profiles classify patients according to severity and urgency, ranging from critical cardiogenic shock (Profile 1) to advanced but stable disease (Profile 7) as shown in Table 1 [1,11].

Acute decompensated HF (ADHF) refers to an acute or subacute worsening of signs and symptoms that require urgent medical therapy or hospitalization. Clinical presentation is usually dominated by pulmonary and systemic congestion, often in the setting of preserved or elevated blood pressure. Perfusion is generally maintained, which distinguishes ADHF from cardiogenic shock [1,11].

CS is the most severe manifestation of acute HF, defined as inadequate cardiac output resulting in systemic hypoperfusion. Although hypotension (systolic blood pressure <90 mmHg or vasopressor dependence) is a hallmark, diagnosis also requires evidence of end-organ compromise such as oliguria, altered mental status, metabolic acidosis, or hepatic and renal dysfunction [4]. Acute myocardial infarction remains the most common precipitating cause of cardiogenic shock, though myocarditis, decompensated chronic heart failure, and postcardiotomy failure are also important etiologies [10]. The underlying pathophysiology may involve decreased contractility (e.g., ischemic cardiomyopathy or myocarditis), increased afterload, or inadequate LV preload due to RV dysfunction. The Society for Cardiovascular Angiography and Interventions (SCAI) shock stages provide a standardized framework for classification, ranging from patients at risk (Stage A) to those with profound circulatory collapse (Stage E) as shown in Table 2 [2].

### 2.2. Neurohormonal Mechanisms in Heart Failure and Cardiogenic Shock

Heart failure and cardiogenic shock are driven by maladaptive neurohormonal activation that promotes adverse remodeling and multi-organ dysfunction. The most important systems are the sympathetic nervous system (SNS), the renin–angiotensin–aldosterone system (RAAS), and arginine vasopressin (AVP), partially counterbalanced by natriuretic peptides, nitric oxide, and other vasodilatory mediators [12,13].

SNS activation occurs early through impaired baroreflex control. Circulating norepinephrine levels increase with severity and predict mortality [14]. While adrenergic drive acutely supports contractility and perfusion, sustained stimulation depletes myocardial norepinephrine, downregulates β-receptors, and disrupts calcium cycling, leading to excess oxygen demand, arrhythmias, and fibrosis [12].

Renal hypoperfusion and adrenergic tone trigger RAAS activation. Angiotensin II and aldosterone initially maintain arterial pressure through vasoconstriction and sodium retention. Chronically, they promote hypertrophy, fibrosis, oxidative stress, and endothelial dysfunction. The ACE2/Ang-(1–7)/Mas receptor axis provides vasodilatory and antifibrotic counterbalance but is insufficient in advanced disease. AVP levels are often inappropriately high in HF, causing V1a-mediated vasoconstriction and V2-mediated water retention with hyponatremia [12].

Counter-regulatory mechanisms oppose these effects but become blunted as disease progresses. Natriuretic peptides normally promote natriuresis, vasorelaxation, and inhibition of RAAS and fibrosis, but their renal actions become ineffective in advanced HF [13]. Endothelial nitric oxide is diminished by oxidative stress, and other vasodilatory mediators such as bradykinin, adrenomedullin, and apelin provide only limited protection. The imbalance between persistent neurohormonal activation and exhausted counter-regulation drives fluid retention, increased afterload, and progressive remodeling—processes that mechanical circulatory support aims to interrupt by restoring perfusion and reducing myocardial stress [15].

### 2.3. Hemodynamic Effects of Mechanical Circulatory Support Devices

MCS devices aim to stabilize hemodynamics, unload the failing ventricle(s), maintain or augment cardiac output, and preserve end-organ perfusion [11,16]. These goals are achieved by assisting or entirely bypassing the native ventricle in propelling blood forward [17]. Depending on clinical needs and the specific ventricle involved, MCS devices can provide either partial or full support and can be used for either temporary or long-term purposes. Short-term MCS options include the intra-aortic balloon pump (IABP), left-sided percutaneous mechanical circulatory assist devices such as Impella (Abiomed, Danvers, MA, USA) and TandemHeart (LivaNova, London, UK), percutaneous right ventricular assist devices such as Protek Duo (LivaNova, London, UK) or Impella RP Flex (Abiomed, Danvers, MA, USA), and veno-arterial extracorporeal membrane oxygenation (VA-ECMO). Long-term MCS options primarily consist of left ventricular assist devices (LVADs) [18]. An overview of hemodynamic characteristics and clinical indications of different MCS devices is provided in Table 3. While the primary goal of MCS is to enhance end-organ perfusion and decrease myocardial oxygen demand, the resulting alterations in hemodynamic parameters compared with normal physiology are illustrated in Table 4 [19]. The following sections outline the mechanisms, physiological effects, and clinical implications of the respective devices.

### 2.4. Device-Specific Physiological Effects and Clinical Management

#### 2.4.1. Intra-Aortic Balloon Pump (IABP)

Mechanism: A balloon catheter placed in the descending aorta inflates during diastole and deflates just before systole to augment diastolic blood pressure and provide systolic unloading [20].

**Figure 1:**
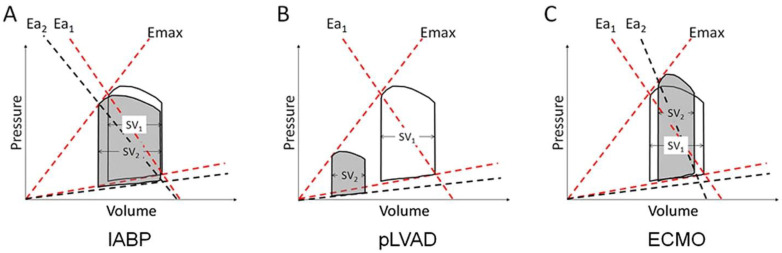
Left Ventricular Pressure-Volume Changes Induced by Mechanical Circulatory Support (MCS) Devices. Baseline cardiac function is shown by non-shaded loops, while changes after device implementation are represented by shaded loops. Emax represents load-independent contractility, indicated by the maximal slope of the end-systolic pressure-volume relationship, and Ea represents arterial elastance. (**A**) Intra-aortic balloon pump (IABP) counterpulsation decreases peak left ventricular (LV) pressures and enhances stroke volume, leading to reduced Ea. (**B**) Percutaneous left ventricular assist devices (pLVADs), such as Impella and TandemHeart, significantly reduce LV pressures and volumes, decreasing cardiac workload without notably altering Emax. (**C**) Veno-arterial extracorporeal membrane oxygenation (VA-ECMO) without ventricular unloading strategy increases LV pressures and reduces stroke volume, thus increasing Ea and ventricular workload. Reprinted from Rihal et al. [10] with permission from Elsevier.

Physiological Effects: IABP provides modest hemodynamic support primarily by increasing diastolic blood pressure, which significantly enhances coronary perfusion pressure. Additionally, the decreased afterload during systole reduces left ventricular end-diastolic pressure (LVEDP) and left ventricular wall stress, potentially enhancing cardiac output (CO) (0.5–1 L/min) and decreasing myocardial oxygen demand [21] as shown in Figure 1. However, clinical studies have shown mixed results. For instance, in patients with chronic heart failure, IABP improved mixed venous oxygen saturation, suggesting increased tissue perfusion [22]. Conversely, in patients with acute heart failure secondary to myocardial infarction, IABP did not significantly improve cardiac output, pulmonary capillary wedge pressure (PCWP), or mean arterial pressure (MAP) [23]. Thus, the hemodynamic benefits of IABP may vary depending on the underlying clinical scenario and patient characteristics.

Clinical Management: The IABP is most effective in patients with partially preserved cardiac function, commonly serving as adjunctive rather than primary support. It has limited efficacy in severe cardiogenic shock when compared to other MCS options [24]. Although no MAP target has been definitively established, maintaining a MAP of approximately 65 mmHg has been suggested as a general guideline for patients with cardiogenic shock requiring temporary MCS [7].

#### 2.4.2. Impella (Left-Sided)

Mechanism: A microaxial catheter-based pump positioned across the aortic valve directly unloads the left ventricle, pumping blood into the ascending aorta.

Physiological Effects: The Impella device reduces LV end-diastolic pressure and volume, which decreases preload and pulmonary congestion because of LV unloading as shown in Figure 1. The Impella also lowers myocardial oxygen demand through reduced wall stress and increases cardiac output (up to approximately 3.5 L/min for Impella CP, 5.0 L/min for Impella 5.0, and 5.5 L/min for Impella 5.5).

Clinical Management: A left-sided Impella is beneficial in severe LV dysfunction, high afterload conditions, or high-risk percutaneous coronary interventions (PCI). It improves coronary and systemic perfusion and may promote myocardial recovery but does not support RV function; thus, caution is needed in biventricular failure [6,25]. Hemodynamic management should target a MAP above 65 mmHg, with careful preload optimization to prevent suction events [26]. Preload optimization can be guided by echocardiography or invasive hemodynamic monitoring, including the calculation of LVEDP, which is available specifically in the Impella CP and 5.5 models with SmartAssist. Afterload must be managed judiciously, potentially using vasodilators if SVR is elevated, which may limit the effectiveness of the device.

#### 2.4.3. TandemHeart

Mechanism: The TandemHeart device, placed transeptally via a transvenous approach, functions as a left atrial-to-femoral arterial bypass circuit, withdrawing oxygenated blood from the left atrium and returning it directly to systemic circulation using an ECMO pump.

Physiological Effects: The TandemHeart device reduces LV preload, wall stress, and LVEDP, as well as PCWP. Unlike ECMO, it offers the advantage of significantly decreasing LV preload through more direct emptying, which is beneficial for patients with pulmonary congestion and edema. However, it does require intrinsic LV ejection and may increase LV afterload. Without adequate unloading strategies, there is a risk of LV distention. The pressure-volume loop typically narrows due to reduced LV end-diastolic volume and increased end-systolic volume [19] as shown in Figure 1.

Clinical Management: The TandemHeart provides substantial support (up to 5 L/min) but requires invasive procedures, such as atrial septostomy, which limits its routine use [27,28].

#### 2.4.4. Right Ventricular Assist Device (RVAD)

Mechanism: RVADs draw blood from the right atrium or ventricle and pumps it into the pulmonary artery, thereby supporting failing right ventricular function (e.g., isolated RV failure, post-cardiotomy RV failure, RV failure post LVAD implantation, or as a bridge to transplantation). Surgical RVAD options include the CentriMag (Abbott Laboratories, Chicago, IL, USA), while percutaneous alternatives include the Protek Duo and the Impella RP. The CentriMag and Protek Duo configurations allow for oxygenator placement for patients with concomitant respiratory failure.

Physiological Effects: RVADs increase pulmonary blood flow, improving LV preload and systemic output if LV function or LV support is adequate. They also reduce central venous pressure (CVP) and thus decrease hepatic and renal congestion. RV unloading decreases RV wall tension and myocardial oxygen consumption [29].

Clinical Management: Patients initially supported with an RVAD may require careful fluid resuscitation to optimize preload, transitioning to diuresis once stable. RV afterload must be optimized by using pulmonary vasodilators such as milrinone [30]. The effects of inhaled pulmonary vasodilators (e.g., epoprostenol or nitric oxide) should be carefully considered in the presence of significant LV dysfunction, as this may adversely impact LV performance. Close monitoring of RVAD flow (typically 3–5 L/min), echocardiographic assessment, and hemodynamic parameters (CVP, pulmonary artery pressures) are critical. Proactive management of potential complications, including hemolysis, thrombosis, bleeding, and infections, is important [29,30].

#### 2.4.5. Veno-Arterial (VA-ECMO)

Mechanism: VA-ECMO withdraws venous blood through a venous cannula, externally oxygenates it, and returns it to systemic circulation via arterial cannulation (commonly the femoral artery in peripheral VA-ECMO), effectively bypassing heart and lung functions [31,32]. Central cannulation, typically performed via the ascending aorta, is used in the surgical settings and provide direct access for arterial return. Axillary artery cannulation is a safe alternative, often used in patients with peripheral vascular disease or limited femoral access. Both central and axillary cannulation deliver antegrade (forward) flow, whereas femoral cannulation produces retrograde flow, with important physiological consequences. Peripheral cannulation via the femoral artery carries the riks of limb ischemia and may require placement of a distal perfusion catheter to ensure adequate limb flow. Close monitoring is important and should include physical examination, Doppler signals, or near-infrared spectroscopy.

Physiological Effects: VA-ECMO significantly increases systemic perfusion and decreases RV preload but concurrently increases LV afterload due to retrograde arterial flow with peripheral cannulation as shown in Figure 1. The increased afterload elevates LVEDP, left atrial pressure, and PCWP, raising the risk of LV distention, higher myocardial oxygen demand, and pulmonary edema. This increased LV afterload, and the risk of LV distension are mitigated when arterial cannulation is performed centrally or axillary rather than femorally. Additionally, the LV preload is not necessarily reduced; the LV can still fill from several sources, including thebesian vessels, bronchial vessels, right heart blood not drained by the venous cannula, or retrograde flow from aortic regurgitation [33]. While inotropic support can provide “chemical unloading”, mechanical adjunctive measures such as IABP, left ventricular Impella, surgical ventricular venting, or atrial septostomy are often necessary to unload the LV [34]. However, data comparing unloading methods are limited; observational studies suggest early Impella unloading may improve outcomes [35], but no randomized trials have definitively established superiority [34]. Despite these potential risks, increased afterload from ECMO can improve coronary perfusion, increase oxygen supply, and correct metabolic derangements due to hypoperfusion, potentially aiding LV function.

Clinical Management: VA-ECMO can be lifesaving in refractory cardiogenic shock or cardiac arrest (extracorporeal cardiopulmonary resuscitation—ECPR—scenarios) [36]. Effective management involves ensuring adequate venous drainage, promptly correcting hypovolemia, and optimizing LV ejection by utilizing inotropes or reducing afterload with vasodilators when clinically appropriate. Maintaining a pulse pressure above 10 mmHg or a CO approximately at least equal to one-fifth of ECMO flow is recommended to facilitate LV ejection, minimizing risks of LV distention and thrombus formation in the aortic root [37]. A significant complication associated with peripheral VA-ECMO is Harlequin syndrome (also known as north–south syndrome), characterized by differential oxygenation [38]. This occurs in the setting of concomitant respiratory failure when poorly oxygenated blood propelled by native LV function mixes with oxygenated retrograde ECMO blood distal to critical proximal aortic branches, leading to hypoxia of cerebral and coronary arteries. Monitoring with a right radial arterial line is recommended to promptly detect this complication. A narrow pulse pressure suggests that the mixing point is closer to the aortic root with lower risk, whereas a larger pulse pressure indicates potential for a more distal mixing point and higher risk. Further, arterial blood gas obtained from a right arterial line would indicate hypoxemia despite effective oxygenation by the ECMO circuit. Management strategies include optimizing ventilation, considering more central arterial cannulation, reducing LV ejection with negative inotropes, or employing hybrid cannulation strategies (e.g., veno-arterial-venous ECMO) [33]. Axillary or central arterial cannulation may improve upper-body oxygenation compared with femoral retrograde flow; however, these strategies alone do not prevent pulmonary edema, which is primarily related to LV distention and elevated afterload [31,32].

#### 2.4.6. Durable Left Ventricular Assist Device (LVAD)

Mechanism: LVADs withdraw blood from the LV and pump it directly into the ascending aorta, thereby unloading the LV, reducing LVEDP, decreasing myocardial oxygen demand, and improving systemic perfusion. The most widely used durable LVAD currently available is the continuous-flow HeartMate 3 (Abbott Laboratories, Chicago, IL, USA) [39].

Physiological Effects: Adequate LVAD function depends on optimized preload (preload dependence). Hypovolemia and/or RV dysfunction may reduce LV and device filling and thus forward flow. At its worst, this may lead to suction events when the ventricle collapses around the device inflow and the pump then cannot flow. Elevated SVR increases the pump work required for forward flow, potentially diminishing LVAD performance (afterload sensitivity) [40]. Continuous-flow LVADs typically produce continuous blood flow without the need for LV ejection, leading to a triangular and leftward-shifted pressure-volume loop as shown in Figure 1 [19]. Increased flow and thus arterial pressure improve oxygenation, myocardial perfusion, and correct metabolic derangements. However, continuous flow often reduces or eliminates aortic valve opening, increasing the risk of blood stasis in the aortic root and potential thrombus formation. Continuous forward flow may also exacerbate aortic regurgitation, creating a feedback loop that decreases systemic output and elevates LV filling pressures. LV unloading reduces myocardial wall stress and oxygen consumption, potentially promoting myocardial recovery [19]. Typically, left atrial and pulmonary pressures decrease, improving pulmonary congestion and dyspnea. Nevertheless, the reduced pulsatility of the aortic pressure waveform may adversely impact baroreceptor reflexes and end-organ perfusion, particularly affecting kidney and brain function.

Clinical Management: The target MAP for LVAD-supported patients is generally 70–80 mmHg [41]. Adequate preload is essential for optimal LVAD function, but attention must be given to avoiding RV failure precipitated by hypervolemia or increased RV afterload. CVP monitoring, ideally maintained below 15 mmHg, is helpful to guide fluid management, diuresis, and inotropic support if needed [42]. Echocardiography plays a key role in assessing LV unloading, aortic valve function, inflow and outflow position, and guiding LVAD speed and flow adjustments, especially in patients with elevated CVP due to positive pressure ventilation.

Afterload should be carefully managed, using vasopressors for reduced afterload and cautious use of vasodilators when afterload is elevated. Maintenance of aortic valve opening is important for reducing thrombotic risks and improving outcomes [43]. Should a suction event occur, management involves reducing pump speed initially, followed by targeted treatment of the underlying cause (e.g., hypovolemia, RV failure, cardiac tamponade) [44]. Individualized anticoagulation plans are necessary based on patient-specific bleeding and thrombosis risks [45]. Clinicians must remain vigilant for complications including gastrointestinal bleeding (associated with acquired von Willebrand deficiency and arteriovenous malformations due to laminar flow), thrombosis, hemolysis, and infections [40].

#### 2.4.7. Biventricular Assist Device (BiVAD)

Mechanism: BiVADs typically consist of combined LVAD and RVAD systems and employed to simultaneously support both the left and right ventricles in patients with severe biventricular failure. Durable BiVAD systems commonly involve two separate surgically implanted centrifugal flow pumps, such as the HeartMate 3, each dedicated to supporting one ventricle. Temporary BiVAD options include a serial surgical RVAD and left-sided Impella device or percutaneous configuration of biventricular catheter-based pumps, like the Impella or ProtekDuo, with the latter providing more rapid deployment and stabilization [46,47].

Physiological Effects: The optimal function of BiVADs requires meticulous coordination of device outputs to ensure balanced ventricular unloading and adequate systemic and pulmonary perfusion. Proper matching of RVAD and LVAD flows is essential; if RVAD flow exceeds LVAD flow, it may precipitate pulmonary overcirculation, which results in pulmonary edema due to increased pulmonary blood volume and elevated pulmonary pressures. Conversely, excessive LVAD flow relative to RVAD output may lead to systemic hypoperfusion and inadequate preload to the left ventricle, compromising end-organ perfusion. Additionally, imbalanced support between the two ventricles can result in interventricular septal shifts, distorting ventricular geometry and adversely impacting ventricular contractility and overall cardiac efficiency.

Clinical Management: Effective BiVAD management thus requires careful coordination of preload optimization, afterload modulation, and device flow coordination. Management of BiVAD-supported patients requires continuous and vigilant monitoring. This involves both invasive hemodynamic assessments (such as pulmonary artery catheters) and non-invasive modalities (echocardiography). Frequent adjustments to device flow settings based on these comprehensive evaluations in a dynamic patient environment are necessary to maintain optimal hemodynamics. Regular echocardiographic assessments help detect complications such as septal malposition, ventricular distension, or cannula malposition, and guide flow adjustments to restore balanced support [48]. Anticoagulation strategies for the major MCS devices are outlined in Table 5 [49,50].

### 2.5. Ventricular-Arterial Coupling, Oxygen Delivery, and Metabolic-Neurohormonal Effects During MCS

Ventricular-arterial coupling refers to the relationship between ventricular contractility and arterial load, a critical determinant of cardiovascular efficiency. MCS significantly modifies this relationship. For example, LVADs typically improve LV-arterial coupling by reducing afterload and enhancing systemic perfusion, thus decreasing myocardial workload and improving ventricular efficiency. Conversely, with RVADs, RV-arterial coupling heavily depends on pulmonary vascular resistance (PVR). Elevated PVR can limit RVAD effectiveness, necessitating adjunct therapies such as pulmonary vasodilators (e.g., inhaled nitric oxide) to optimize RV support and overall hemodynamic stability [19].

MCS devices enhance tissue oxygen delivery (DO_2_) primarily through improved systemic perfusion. In shock states, impaired DO_2_ leads to anaerobic metabolism and subsequent lactic acidosis. Restoration of adequate DO_2_ by MCS facilitates aerobic metabolism and lactate clearance. Continuous monitoring of markers such as lactate, central venous oxygen saturation (ScvO_2_), and mixed venous oxygen saturation (SvO_2_) is crucial for guiding clinical management and assessing therapeutic effectiveness [51].

Persistent neurohormonal activation (e.g., RAAS, sympathetic nervous system) and microcirculatory dysfunction may continue despite the normalization of macrocirculatory parameters (e.g., MAP and CO) with MCS. MCS may improve end-organ function by increasing perfusion. However, continuous-flow LVADs can induce non-physiologic flow patterns, potentially affecting endothelial function, impairing baroreceptor reflexes, autoregulation and renal perfusion [51].

**Figure 2:**
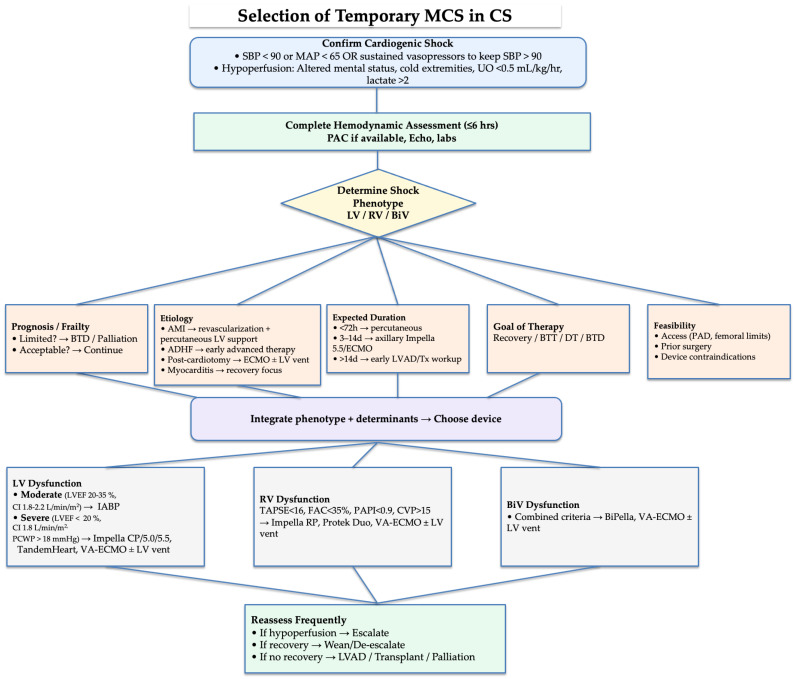
Flow chart for temporary MCS selection in cardiogenic shock. Cardiogenic shock is defined as SBP <90 mmHg or MAP <65 mmHg, or sustained need for inotropes/vasopressors to maintain SBP >90 mmHg, with evidence of hypoperfusion. Device selection is guided first by shock phenotype (LV, RV, or biventricular dysfunction), and then integrated with additional determinants including prognosis/frailty, underlying etiology (AMI, ADHF, post-cardiotomy, myocarditis), expected duration of support, therapeutic goal (recovery, bridge-to-transplant, destination, or bridge-to-decision), and feasibility (vascular access, prior surgery, device-specific contraindications). LV: Left Ventricle, RV: Right Ventricle, LVEF: Left Ventricular Ejection Fraction, CI: Cardiac Index, PCWP: Pulmonary Capillary Wedge Pressure, TAPSE: Tricuspid Annular Plane Systolic Excursion, FAC: Fractional Area Change, PAPI: Pulmonary Artery Pulsatility Index, CVP: Central Venous Pressure, IABP: Intra-Aortic Balloon Pump, ECMO: Extracorporeal Membrane Oxygenation. PAPi = (PA systolic pressure—PA diastolic pressure)/CVP.

**Figure 3:**
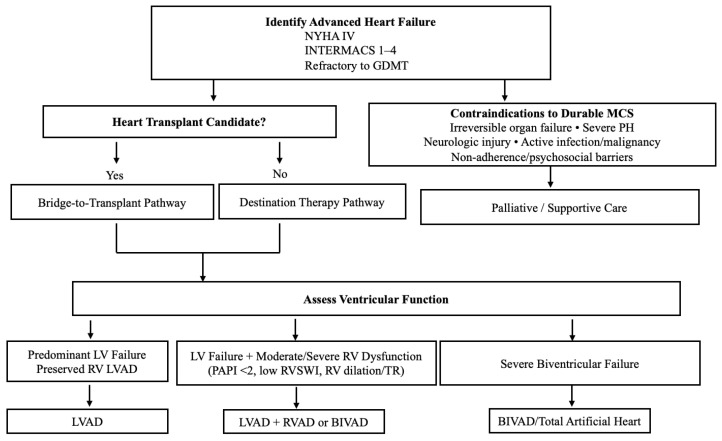
Flowchart for Selection of Long-Term MCS. Long-term MCS selection pathways for advanced heart failure (NYHA IV, INTERMACS 1–4). Options include bridge-to-transplant, destination therapy with LVAD/BiVAD, or palliative care, depending on transplant eligibility, ventricular function, and contraindications.

Applying these physiological principles to clinical decision-making is important. Figure 2 provides a practical algorithm to guide the selection of temporary MCS devices based on the severity and type of ventricular dysfunction. An overview of long-term MCS selection pathways is provided in Figure 3.

### 2.6. Clinical Algorithms for MCS Selection

The application of MCS in clinical practice requires structured decision pathways. The choice of temporary MCS in cardiogenic shock needs to consider factors such as ventricular involvement, prognosis, anticipated duration of support, and feasibility, as shown in Figure 2 [7,18]. Pathways for durable MCS in advanced heart failure must take into account eligibility for transplantation, contraindications to long-term support (e.g., severe comorbidities, irreversible end-organ dysfunction, or active infection), and therapeutic options such as bridge-to-transplant, destination therapy, or palliative care, as shown in Figure 3 [52,53].

## 3. Clinical Management Strategies and Evidence Based Use of MCS Devices

### 3.1. IABP

First introduced in 1968 [54], the IABP has been widely used for cardiogenic shock and perioperative support [55,56]. It is safe, cost-effective, and percutaneously inserted, with major complications occurring in <1% of cases [56,57]. Although initially recommended as Class I therapy, the IABP-SHOCK II trial demonstrated no survival benefit in myocardial infarction–related cardiogenic shock [58,59], leading to guideline downgrading to Class III (no benefit) [60,61]. A meta-analysis suggested potential benefit when used preoperatively in high-risk CABG patients [62], but overall clinical practice has shifted toward newer MCS devices.

### 3.2. Impella

The Impella, derived from the Hemopump (1985), received CE approval in 2005 and FDA approval in 2008 [63]. It provides hemodynamic support in high-risk PCI, cardiogenic shock, LV failure, RV failure (Impella RP Flex), and perioperative settings (e.g., during ventricular tachycardia ablation, high-risk PCI, post-cardiopulmonary bypass, or post-transcatheter aortic valve replacement). It can also serve as a bridge to transplant or for LV venting during VA-ECMO [64].

Smaller devices (e.g., Impella CP) are placed percutaneously, while Impella 5.5 requires surgical transaxillary implantation, allowing longer-term support and patient mobility. Complications include vascular injury (7–8%), bleeding (15%), hemolysis (~10%), and, with the transaxillary approach, up to 17% serious complications and ~50% in-hospital mortality in high-risk patients [65,66].

Evidence for outcomes is mixed. The Impella 2.5 provides limited hemodynamic support and has not shown a survival benefit in cardiogenic shock [66]. In contrast, larger devices (5.0/5.5) demonstrate improved outcomes, with ~70% one-year survival in post-cardiac surgery shock [67,68]. The DanGer Shock trial reported a 12.7% absolute reduction in 180-day mortality with Impella CP in STEMI-related shock, although this was associated with higher complication rates, including bleeding, limb ischemia, and the need for renal replacement therapy [6]. Earlier studies such as PROTECT II found no significant advantage of Impella 2.5 over IABP in high-risk PCI [69], and subsequent analyses also showed no 30-day mortality difference between Impella 2.5 and IABP in myocardial infarction–related shock, despite higher complication rates with Impella [70]. To date, no direct comparative trials exist between IABP and larger Impella devices.

### 3.3. VA-ECMO

VA-ECMO rapidly stabilizes patients with cardiac arrest, refractory cardiogenic shock, post-cardiotomy failure, or acute intra-procedural collapse, serving mainly as a temporary bridge to recovery or definitive therapy [71]. Complications of prolonged use include bleeding, systemic inflammation, limb ischemia, thrombotic events, and LV distension, often requiring adjunctive unloading with IABP or Impella [72,73].

Evidence for ischemia-related cardiogenic shock is mixed. The EURO SHOCK trial (terminated early due to COVID-19) suggested possible survival benefit, though with increased bleeding and vascular events [74]. In contrast, the larger ECLS-SHOCK trial showed no improvement in 30-day or 12-month mortality and confirmed higher complication rates [75]. A recent meta-analysis likewise found no overall survival benefit from early percutaneous MCS in AMI-related shock, except in a subgroup of STEMI patients without hypoxic brain injury [9,72,73].

Registry and observational data highlight potential benefits in specific populations: survival of 34–100% in pulmonary embolism [71], 61% in myocarditis [76], and procedural stability during ventricular tachycardia ablation [77]. Meta-analyses comparing Impella and VA-ECMO show similar mortality but fewer bleeding and stroke complications with Impella [78]. Combined use of Impella and VA-ECMO may improve short-term outcomes but is associated with higher risks of hemolysis, ischemia, and renal failure requiring dialysis [79].

### 3.4. Durable LVAD

Durable LVADs are indicated for advanced heart failure (NYHA III–IV) refractory to medical therapy, serving as a bridge to transplant, bridge to candidacy, or destination therapy [80,81]. The MOMENTUM 3 trial reported HeartMate 3 survival rates of 79% at 2 years and 58% at 5 years [82], consistent with ELEVATE registry findings (74.5% and 63.3%) [83]. Common complications include bleeding, driveline infections, and progressive aortic regurgitation (≈30%) [84]. Right heart failure occurs in 20–40% of patients, requiring RV support in ~5% [85]. While pump thrombosis is less frequent with HeartMate 3, gastrointestinal bleeding, arrhythmias, and stroke remain important concerns [84].

### 3.5. BIVAD

Patients with chronic biventricular failure remain difficult to manage, and evidence is limited. Off-label use of dual HeartMate 3 pumps has emerged as a strategy for biventricular support. In a multicenter series (14 patients), mean support duration was 266 days, with most discharged home [46]. A larger cohort (38 patients) reported one-third mortality during support, while nine patients were bridged to transplant and 18 maintained on support for ~5 months [86]. Complications included device malfunction (25%), bleeding, neurologic, and renal dysfunction.

A concise summary of the key evidence and clinical indications for the MCS devices discussed is presented in Table 6.

## 4. Monitoring During MCS

### 4.1. Pulmonary Artery Catheter (PAC)

PAC monitoring can provide valuable hemodynamic information in patients with cardiogenic shock requiring MCS by helping to categorize the type and severity of shock, guide escalation or de-escalation of support, and assess adequacy of resuscitation [87,88]. Randomized data establishing a treatment algorithm based on PAC findings in cardiogenic shock are lacking, and much of the current evidence is observational. In one observational study, PAC insertion prior to MCS initiation was associated with reduced mortality; however, the strongest predictors of outcome were mean arterial pressure and central venous pressure, which are not specific to PAC [89]. More comprehensive hemodynamic profiling (including right atrial pressure, pulmonary artery pressures, pulmonary capillary wedge pressure, cardiac output, and PA oxygen saturation) showed potential prognostic value, but the overall role of PAC in guiding therapy remains uncertain and should be interpreted with caution [89]. In patients already on VA-ECMO, increased PA pulse pressure compared to pre-ECMO values has been correlated with improved survival, likely reflecting preserved RV function [90].

Ongoing prospective trials such as PACCS (ClinicalTrials.gov Identifier: NCT05485376)**,** which is testing whether early PAC-guided management in cardiogenic shock due to acutely decompensated heart failure reduces in-hospital mortality, may further clarify the role of PAC monitoring. Importantly, this trial excludes patients receiving short-term mechanical support and does not prespecify treatment protocols.

### 4.2. Continuous Cardiac Output and SvO_2_

Continuous monitoring of CO and SvO_2_ using a PAC provides critical information on systemic perfusion and tissue oxygenation during MCS. Accurate assessment of SvO_2_ helps evaluate the balance between DO_2_ and O_2_ consumption, guiding adjustments in device settings and therapeutic management. Specifically, in patients supported with VA ECMO, SvO_2_ measurements accurately reflect systemic perfusion status and are routinely used to monitor the adequacy of circulatory support [91].

### 4.3. Echocardiography

Echocardiography, including transthoracic (TTE) and transesophageal (TEE) approaches, is critical in managing patients with MCS, providing real-time assessment of cardiac anatomy, ventricular function, device positioning, and hemodynamic status. TTE is typically the initial modality due to its non-invasive nature and convenience for rapid bedside assessment; however, its image quality can be limited by factors such as body habitus and acoustic shadowing from implanted devices. TEE is frequently used in the perioperative and intensive care settings for accurate device placement, real-time monitoring, and early detection of complications, especially when TTE imaging quality is suboptimal [92].

Further, echocardiography has a specific utility for individual MCS devices [93]. For Impella devices, it is used to confirm appropriate positioning across the aortic valve, verify effective left ventricular unloading, and identify complications such as valve damage, ventricular wall injury, or device migration. For TandemHeart and ProtekDuo devices, echocardiography, particularly TEE, is used to guide critical steps such as transseptal puncture (TandemHeart), optimal cannula positioning, and effective ventricular decompression. It is also used to confirm correct inflow cannula placement in the left atrium (TandemHeart) or the pulmonary artery (ProtekDuo).

In patients receiving VA-ECMO, particularly in the setting of ECPR, echocardiography is valuable for excluding contraindications such as aortic dissection and severe aortic insufficiency, before implantation. Post-initiation, it is important for detecting complications, including left ventricular distension, inadequate ventricular ejection, cannula malposition, and thrombus formation; however, imaging can be challenging due to acoustic shadowing from ECMO cannulas [94]. During weaning from VA-ECMO, echocardiographic parameters predictive of successful decannulation include increased aortic valve opening, improved left ventricular ejection fraction (LVEF ≥20–25%), left ventricular outflow tract velocity-time integral (LVOT VTI >10 cm), adequate RV function (RV ejection fraction ≥25%, and Tricuspid Annular Plane Systolic Excursion [TAPSE] ≥19 mm) [95]. Therefore, echocardiography is indispensable for safe and effective placement, management, and weaning of patients receiving MCS.

### 4.4. Arterial Waveform Analysis

Arterial waveform analysis provides valuable hemodynamic information during MCS. In the context of IABP management, waveform analysis helps evaluate ventricular unloading and device performance. In patients receiving Impella support, internal waveform data, including motor-current and pressure signals from the device controller, assist with correct positioning and early detection of complications such as suction events, although standard arterial pressure monitoring remains necessary.

During VA-ECMO support, arterial waveform analysis is especially beneficial for monitoring for recovery and during weaning phases as the reappearance of pulsatile waveforms typically indicates improved myocardial function and sufficient native CO [19]. Commercially available monitoring systems analyze arterial waveforms from standard arterial lines and derive parameters such as SV, CO, stroke volume variation (SVV), and pulse pressure variation (PPV). Despite their established utility in the perioperative and critical care environments, the accuracy and applicability of these devices during MCS are still unknown but likely limited by reduced arterial pulsatility and variable SVR [96].

### 4.5. End-Tidal Carbon Dioxide (EtCO_2_)

End-Tidal Carbon Dioxide (EtCO_2_) monitoring provides a non-invasive assessment of pulmonary blood flow and indirectly reflects native CO, making it particularly valuable during ECMO support and weaning phases. An increase in EtCO_2_ suggests improved native cardiac function, as increased pulmonary blood flow results in greater CO_2_ clearance. A study involving 37 VA ECMO patients demonstrated that an EtCO_2_ rise of at least 5 mmHg over 12 h reliably predicted successful ECMO weaning [97]. Thus, EtCO_2_ monitoring serves as a valuable adjunct to invasive hemodynamic assessment, facilitating clinical decision-making for optimal timing of ECMO weaning.

### 4.6. Device-Specific Monitoring

#### 4.6.1. VA-ECMO Parameters: Flow Rates and Oxygenation Indices

ECMO flow serves as a surrogate for CO, with recommended target flows between 50 and 75 mL/kg/min for adult patients. Larger patients may require additional venous drainage cannulas to achieve adequate perfusion. Gas exchange during ECMO relies on a gradient created by fresh gas, termed “sweep gas,” which removes CO_2_ and delivers O_2_. Oxygenation improves with increased blood flow or higher fractions of delivered oxygen, independent of sweep gas flow, because more blood is exposed to the oxygenator membrane. Conversely, CO_2_ elimination directly correlates with sweep gas flow; increasing sweep gas enhances CO_2_ removal, analogous to increasing minute ventilation in patients without ECMO support [98].

#### 4.6.2. LVAD Parameters (HeartMate 3): Pump Speed, Flow, and Pulsatility Index

HeartMate 3 is the dominant device for durable LVAD support. Its pump speed typically ranges between 3000 and 9000 revolutions per minute (RPM), but it is commonly maintained above 4000 RPM. Though a continuous flow device, the HeartMate 3 employs intrinsic pulsatility through periodic decreases and increases in device speed every two seconds to improve flow dynamics and decrease the risk of thrombosis. LVAD flow depends on both RPM and afterload; flow decreases as afterload increases. By contrast, reduced preload may trigger a “low-speed limit,” known as a “suction event.”

Flow estimates are derived from pump power consumption; due to the linear relationship between flow and power at a fixed speed, factors like pump thrombosis that increase power can produce falsely elevated flow readings. The pulsatility index (PI) reflects increased pump flow during ventricular systole and typically remains below 10 in optimal conditions. Lower PI values indicate reduced ventricular activity, while higher values suggest increased pulsatility and thus improved cardiac function. A stable PI at rest is expected, and an abrupt PI drop can signal reduced preload [40].

## 5. Special Considerations

### 5.1. Physiological Adaptation During Weaning from MCS

**Figure 4:**
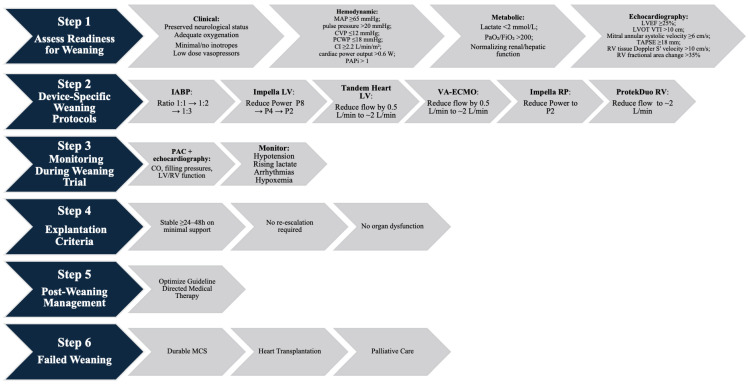
Algorithm from Weaning from temporary MCS. This algorithm outlines a stepwise process for weaning temporary MCS. Readiness is assessed across clinical, hemodynamic, metabolic, and echocardiographic domains, followed by device-specific weaning strategies, structured monitoring, and predefined explantation criteria. Importantly, patients do not need to meet all criteria individually before beginning weaning or device discontinuation; rather, the overall clinical picture should guide the decision. Post-weaning management focuses on guideline-directed therapy initiation, while failed weaning prompts transition to durable MCS, transplantation, or palliative care.

One of the key physiologic changes in MCS for any indication is ventricular offloading to decrease wall stress and myocardial oxygen demand [99]. This is theoretically more notable in central cannulation and temporary percutaneous VADs, where unloading is much more complete due to more proximal drainage [100]. In contrast, patients on peripheral VA ECMO may face increased LV afterload, particularly at higher flows, due to the retrograde nature of perfusion [100]. As such, the weaning from MCS innately requires reloading of a recently stressed ventricle, and thus liberation from MCS hinges on tolerance of this process, particularly for the right ventricle [100]. There are currently no standardized protocols regarding the timing or strategies for tMCS weaning. Assuming the underlying cause has been treated, improved, or resolved, weaning should ideally be initiated within 48–72 h of MCS initiation with daily reassessment. Figure 4 illustrates a structured algorithm for temporary MCS weaning.

Some patients status post LVAD placement have functional myocardial recovery and reverse structure remodeling due to the long-term offloading of the left ventricle allowing the previous “stretch” to be undone [101]. The RESTAGE-HF trial demonstrated that nearly half of patients achieved improvement such that LVAD explantation was accomplished and recovered function was sustained for a year [102]. This physiologic change is the premise behind BTR pathways previously discussed. The molecular pathways to explain this are being investigated [101]. Recently, this finding was explored in temporary MCS modalities including prolonged use of an Impella device via the PROPELLA trial which identified disease-modifying effects of Impella which predisposed myocardial recovery in patients with fulminant myocarditis [103].

### 5.2. Ethical Consideration in MCS

MCS is often initiated rapidly in acutely decompensating patients, forcing clinicians, patients, and families to make life-or-death decisions under conditions of uncertainty and incomplete information. When caring for patients receiving MCS, clinicians should apply the principles of clinical bioethics and carefully balance the potential benefits of support against the risks of futility, prolonged suffering, and poor quality of life.

- Autonomy: obtaining informed consent for emergent MCS is ethically complex because most patients are critically ill and unable to participate in decision-making, placing the burden on families or other legal surrogates [104]. In some situations—such as the operating room or catheterization laboratory—there is no time to obtain consent before device placement. In these cases, early involvement of palliative care or ethics consultation may be appropriate to ensure that patients’ wishes and values are respected [105].
- Beneficence: while MCS can provide life-saving physiologic support, it does not guarantee recovery or meaningful long-term survival. For example, only 41% of adults supported with VA-ECMO after cardiotomy survive to hospital discharge, with survival decreasing to 34% after CABG and 30% after aortic surgery [106]. Short-term microaxial pumps (e.g., Impella) have shown a 25–35% reduction in six-month mortality in post-STEMI cardiogenic shock [6,107]. Conversely, early VA-ECMO initiation in severe cardiogenic shock has not consistently improved survival [75,108,109]. These data highlight the need to weigh potential benefits against the risks of prolonged debility and suffering, with early family discussions and palliative input helping align treatment with patient values.
- Nonmaleficence: determining when MCS becomes futile is one of the most challenging aspects of care. Devices such as VA-ECMO may create a perception of ongoing hope even when recovery is unlikely, leading families to resist withdrawal. The absence of consensus guidelines further complicates decisions and can contribute to moral distress among care teams. Multidisciplinary discussions and ethics consultations are essential to navigate these situations with compassion and clarity [110].
- Justice and Equity: MCS requires substantial resources, specialized staff, and prolonged ICU care, raising concerns about justice and equity [111]. Access is uneven, with only 67% of the U.S. population living within reach of an ECMO-capable center by ground transportation, while the remaining 33% have no such access, including all of Puerto Rico and the states of Wyoming, North Dakota, and Alaska [112]. Triage tools such as the SAVE score may prioritize those most likely to benefit but risk excluding marginalized patients who already face barriers to timely care [113]. Ensuring equitable distribution of MCS, therefore, requires balancing clinical benefit with fairness and transparency.

## 6. Emerging Role of Artificial Intelligence in MCS

Artificial Intelligence (AI) refers to the ability of computer systems to learn from data and perform tasks that typically require human intelligence, such as decision-making and problem-solving. Machine Learning (ML), a subset of AI, involves training these systems to recognize complex patterns and accurately predict outcomes directly from data. Recently, AI prediction models have significantly advanced cardiac care, with applications in echocardiography interpretation, electrocardiogram (EKG) analysis, and heart transplant outcome predictions [114]. In the context of MCS, AI can utilize extensive clinical datasets to identify optimal candidates, anticipate potential complications such as right ventricular failure, and determine the most appropriate type of mechanical support. Post-implantation, AI-driven algorithms can dynamically adjust MCS parameters (e.g., LVAD speed, flow rates, pulsatility index) based on real-time patient data, potentially optimizing clinical management [115,116]. These concepts remain largely proof-of-concept, demonstrating feasibility but not yet established in clinical practice.

Some applications are beginning to approach clinical relevance. Recent studies using Bayesian and decision tree models derived from the INTERMACS registry have predicted short- and long-term outcomes following LVAD implantation [117]. Similarly, advanced ML techniques, such as convolutional neural networks and decision tree model, have been applied to echocardiographic analysis for assessment of RV function, improving patient selection and perioperative planning [118,119]. Additionally, ML models analyzing electronic health records and wearable devices have shown potential for early detection of clinical deterioration, facilitating timely interventions, and reducing hospital readmissions [120]. These applications represent important translational steps, though widespread clinical adoption will require prospective validation.

Despite these promising advances, significant challenges remain, including issues related to data integration, model interpretability, and resource availability. At present, the majority of AI work in MCS should be considered exploratory. Continued research and rigorous clinical trials are needed to refine these technologies, validate their effectiveness, and ultimately integrate them safely into routine patient care.

## 7. Conclusions

MCS is a rapidly evolving field requiring a thorough understanding of its cardiovascular interactions and careful selection of monitoring strategies. Given the complexity of MCS devices, individualized patient management, accurate interpretation of monitoring data, and attention to ethical considerations are essential. Future research should focus on addressing device-specific limitations, clarifying their clinical utility, refining monitoring methods, and ensuring safe integration into practice. Emerging technologies like AI require further validation before routine clinical use.

## Figures and Tables

**Table 1:** INTERMACS Profiles for Advanced Heart Failure.

Profile	Description
1—Critical shock	“Crash and burn,” immediate support required
2—Progressive decline	Worsening despite inotropes
3—Stable but inotrope dependent	Continuous inotrope requirement
4—Frequent hospitalizations	Recurrent decompensation episodes
5—Exertion limited	Stable but severe limitation
6—Stable, exertion limited	Symptoms with daily activity
7—Advanced but stable	NYHA III, early referral stage

**Table 2:** SCAI Cardiogenic Shock Stages.

Stage	Description
A—At risk	HF/ACS, stable, no hypoperfusion
B—Beginning	Hypotension/tachycardia, no hypoperfusion
C—Classic	Hypoperfusion requiring inotropes/vasopressors
D—Deteriorating	Worsening shock despite therapy
E—Extremis	Circulatory collapse, refractory arrest/shock

**Table 3:** Overview of Hemodynamic Characteristics and Clinical Indications of Mechanical Circulatory Support Devices.

Device	Flow Support	LV Unloading	RV Support	Invasiveness	Indication
IABP	Low (0.5–1 L/min)	Partial	No	Low	Cardiogenic shock
Impella	Moderate (2.5–5.5 L/min)	Yes	No	Moderate	Severe LV failure, cardiogenic shock
TandemHeart	Moderate (up to 5 L/min)	Yes	No	High	Cardiogenic shock
VA-ECMO	High (3–5+ L/min)	No (↑Afterload)	Yes	Low (Peripheral)/High (Central)	Profound shock/cardiac arrest (ECPR)
LVAD	High (up to 10 L/min)	Yes	No	High	End-stage LV failure, bridge to transplant
RVAD	High (up to 5 L/min)	No	Yes	High	RV failure, post-LVAD RV dysfunction

Abbreviations: LV: Left Ventricle; RV: Right Ventricle; ECPR: Extracorporeal Cardiopulmonary Resuscitation. Invasiveness grading: Low = simple percutaneous access (e.g., IABP); Moderate = percutaneous with larger-bore or transvalvular access (e.g., Impella); High = surgical implantation or procedures requiring atrial septostomy, sternotomy, or extracorporeal circuit cannulation (e.g., TandemHeart, VA-ECMO, durable LVAD, RVAD).

**Table 4:** Overview of Hemodynamic Parameters: Normal Heart versus MCS.

Parameter	Normal Heart	With MCS
Cardiac Output	Dependent on LV contractility, preload, afterload, and heart rate	Enhanced or maintained by device flow
LV End-Diastolic Volume	Reflects preload and ventricular filling	Generally decreased due to ventricular unloading (except increased with VA-ECMO if inadequate decompression)
LV Afterload	Determined by systemic vascular resistance (SVR)	Typically reduced (Impella, IABP); increased with VA-ECMO
Mean Arterial Pressure	Derived from cardiac output × systemic vascular resistance	Often supported artificially independent of native cardiac function
Coronary Perfusion	Depends on diastolic blood pressure and coronary vascular resistance	Improved with IABP; may worsen with VA-ECMO due to increased LV afterload and ventricular distension

**Table 5:** Anticoagulation Strategies Across MCS Devices.

Device	Preferred Anticoagulant	Target Range	Notes
IABP	UFH (variable use)	aPTT 50–70 s (if used)	Often none unless prolonged use or reduced support ratio
Impella	Purge: UFH (traditional) or sodium bicarbonate (increasingly used) Systemic: UFH	Anti-Xa 0.2–0.4 U/mL; aPTT 50–80 s	Bivalirudin if HIT
VA-ECMO	UFH	Anti-Xa 0.3–0.7 U/mL; aPTT 1.5–2.5 × baseline	Alternatives: bivalirudin, argatroban
RVAD	UFH (purge if motor-driven) or systemic UFH	Individualized (often aPTT 60–80 s)	Strategy depends on device type; direct thrombin inhibitors if HIT
Durable LVAD	UFH → Warfarin (±antiplatelet)	INR 2–3	Individualized to bleeding/thrombotic risk

**Table 6:** Evidence and Clinical Indications for MCS Devices.

Device	Key Evidence/Trials	Clinical Settings	Strength of Evidence
IABP	IABP-SHOCK II (no mortality benefit) [58]; meta-analyses suggest perioperative benefit (e.g., pre-CABG) [62]	Ischemic shock (limited role), perioperative stabilization, high-risk PCI	Weak–moderate
Impella	PROTECT II (no benefit vs. IABP in PCI) [69]; DanGer Shock (12.7% mortality reduction at 180 days with Impella CP) [6]; registry data favor larger devices (5.0/5.5) [68]	High-risk PCI, AMI-related CS, perioperative support, LV venting on VA-ECMO	Moderate
VA-ECMO	EURO SHOCK (suggested benefit, early termination) [74]; ECLS-SHOCK (no survival benefit, ↑ complications) [75]	Profound cardiogenic shock, cardiac arrest/ECPR, post-cardiotomy failure, myocarditis, pulmonary embolism with shock	Moderate
Durable LVAD	MOMENTUM 3 (79% 2-year survival, 58% at 5 years) [82]; ELEVATE registry (similar outcomes) [83]	Advanced HF refractory to GDMT; bridge to transplant, bridge to candidacy, destination therapy	Strong
BiVAD	Small multicenter series (dual HeartMate 3 pumps, ~266 days support); registry cohorts show ~1/3 mortality, some bridged to transplant	Severe biventricular failure, bridge to transplant	Weak

## Data Availability

Not applicable.

## Abbreviations

The following abbreviations are used in the manuscript:

AI	Artificial Intelligence
BiVAD	Biventricular Assist Device
CO	Cardiac Output
CS	Cardiogenic Shock
CVP	Central Venous Pressure
DO_2_	Oxygen Delivery
ECPR	Extracorporeal Cardiopulmonary Resuscitation
FAC	Fractional Area Change
HF	Heart Failure
HR	Heart Rate
IABP	Intra-Aortic Balloon Pump
LV	Left Ventricle
LVAD	Left Ventricular Assist Device
LVEDP	Left Ventricular End-Diastolic Pressure
MAP	Mean Arterial Pressure
MCS	Mechanical Circulatory Support
ML	Machine Learning
PAC	Pulmonary Artery Catheter
PAPi	Pulmonary Artery Pulsatility Index
PCWP	Pulmonary Capillary Wedge Pressure
PI	Pulsatility Index
PVR	Pulmonary Vascular Resistance
RAAS	Renin–Angiotensin–Aldosterone System
RPM	Revolutions per Minute
RV	Right Ventricle
RVAD	Right Ventricular Assist Device
SV	Stroke Volume
SVR	Systemic Vascular Resistance
SvO_2_	Mixed Venous Oxygen Saturation
TAPSE	Tricuspid Annular Plane Systolic Excursion
TEE	Transesophageal Echocardiography
TTE	Transthoracic Echocardiography
UFH	Unfractionated Heparin
VA-ECMO	Veno-Arterial Extracorporeal Membrane Oxygenation
